# Analysis of XX *SRY*-Negative Sex Reversal Dogs

**DOI:** 10.3390/ani10091667

**Published:** 2020-09-16

**Authors:** Sara Albarella, Lisa De Lorenzi, Elena Rossi, Francesco Prisco, Marita Georgia Riccardi, Brunella Restucci, Francesca Ciotola, Pietro Parma

**Affiliations:** 1 Department of Veterinary Medicine and Animal Production, University of Naples Federico II, via Delpino 1, 80137 Naples, Italy; francesco.prisco@unina.it (F.P.); brunella.restucci@unina.it (B.R.); francesca.ciotola@unina.it (F.C.); 2 Department of Agricultural and Environmental Sciences, Milano University, via Celoria 2, 20133 Milan, Italy; lisa.delorenzi@unimi.it (L.D.L.); pietro.parma@unimi.it (P.P.); 3 Department of Molecular Medicine, Pavia University, via Forlanini 12, 27100 Pavia, Italy; elena.rossi@unipv.it; 4 Experimental Zooprophylactic Institute of Southern Italy, via Salute 2, 80055 Portici, Italy; maritageorgia.riccardi@izsmportici.it

**Keywords:** bitch, disorder of sex development (DSD), copy number variation (CNV), *SOX9*

## Abstract

**Simple Summary:**

The study of XX *SRY*-negative sex reversal cases is of great interest because testicular tissue develops in these subjects in the absence of *SRY* gene, thus allowing us to deepen the knowledge of all the other genes involved in the development of testes and the structures related to the male reproductive apparatus. This paper reports the results of the study of four new canine disorders of sex development (DSDs) XX *SRY*-negative cases in which 11 copy number variations (CNVs) are observed, five of which are never described.

**Abstract:**

Impaired fertility associated with disorders of sex development (DSDs) due to genetic causes in dogs are more and more frequently reported. Affected dogs are usually of specific breeds thus representing a cause of economic losses for breeders. The aim of this research is to report the clinical, cytogenetic and molecular genetic findings of four XX *SRY*-negative DSD dog cases. All the subjects showed a female aspect and the presence of an enlarged clitoris with a penis bone. Morphopathological analyses performed in three of the four cases showed the presence of testes in two cases and ovotestis in another. Conventional and R-banded cytogenetic techniques were applied showing that no chromosome abnormalities were involved in these DSDs. CGH arrays show the presence of 11 copy number variations (CNVs), one of which is a duplication of 458 Kb comprising the genomic region between base 17,503,928 and base 17,962,221 of chromosome 9 (CanFam3 genome assembly). This CNV, confirmed also by qPCR, includes the promoter region of *SOX9* gene and could explain the observed phenotype.

## 1. Introduction

The occurrence of disorders of sex development (DSDs) is an emerging problem in domestic animals due to the reproductive inability of the affected animals and the medical expenses associated with treatment of the pathology; thus, their early identification will confer a big benefit in animal breeding [1,2]. Disorders of sexual development are congenital conditions in which chromosomal, gonadal, or anatomical sex development do not develop in a coordinated way and cause a failure in the reproductive ability of the carrier. In mammals, the sexual development of an embryo is determined at fertilization. The XY zygotes contain the *SRY* gene, located on the Y chromosome, which initiates a genetic and hormonal pathway that will lead first to the formation of male gonads, the testes, and then to an internal and external male phenotype. The XX zygotes instead develop the female gonads, the ovaries, and therefore an internal and external female phenotype [3]. The pathway of sexual development is very complex, perhaps second only to cognitive development, and involves a large number of genetic and hormonal factors. The failure in the functioning of only one of these factors can lead to the development of an abnormal sexual phenotype. 

There are several types of DSD disorders and a first distinction takes into consideration what disagreement there is between the three stages of determining sex. In pseudo-hermaphrodite phenotypes there is a mismatch between gonadal sex (testes and ovaries) and phenotypic sex (male and/or female). Often, but not always, these phenotypes are caused by errors in the functioning of hormones. Sex reversal (SR) phenotypes are characterized by a discrepancy between chromosomal sex (XY/XX) and gonadal development (testes/ovaries) [4]. The presence or the absence of a *SRY* gene with a normal sequence is checked in these cases as this gene is the key regulator of gonadal development. Therefore, there are four conditions: XX male *SRY*-negative, XX male *SRY*-positive, XY female *SRY*-negative, and XY female *SRY*-positive. In domestic animals, sex reversal disorders have been described in pig [5], goat [6], sheep [7], roe deer [8], llama [9], cattle [10], buffaloes [11], horse [12,13], cat [14], dog [15,16] and ferret [17].

The pathological phenotypes observed in XX-*SRY*-negative individuals are some of the most interesting. In fact, despite the absence of the *SRY* gene, testes develop from primordial gonads which will then give rise to a male phenotype. 

In dogs, this disorder occurs with a not negligible frequency and consequently, in addition to representing damage to breeders, it also represents an excellent animal model for studying this developmental anomaly. 

From a genetic point of view, several genomic regions have been identified as responsible for the onset of this particular phenotype. *SOX9* is probably the most involved gene: (i) gain-of-function (GoF) mutation including at least a small part of the *SOX9* promotor region, called *RevSex* (reviewed by Vetro et al. [18]) leads to a development of a XX-SR phenotype; (ii) an enhancer, *Enh13*, responsible for activating *SOX9* in XY mouse gonads has recently been identified [19]. In dogs, some cases have been associated with GoF mutations in the *SOX9* gene [20]. In addition to *SOX9*, three other possible causes have been identified: i. *RSPO1* loss-of-function (LoF) mutations [21]; ii. deletion of the genomic region including the *FOXL2* gene promotor [22] and iii. *SOX3* duplications [23]. None of these last three causes have been identified in XXSR dogs.

Furthermore, all these genomic alterations explain only a limited percentage of cases of sex reversal dogs, and therefore many cases are still to be explained and could be useful for the identification of new causative genes related to this condition.

In this work, the initial characterization is presented (a fundamental step to be able to proceed further to more in-depth genomic analyzes) of four new cases of canine XX *SRY*-negative DSD. 

## 2. Material and Methods

### 2.1. Ethical Statement

All the procedures used in this research were approved by the Ethical Animal Care and Use Committee of the University of Naples Federico II (PG2020/0014495 del 07/02/2020).

### 2.2. Cases

**Figure 1:**
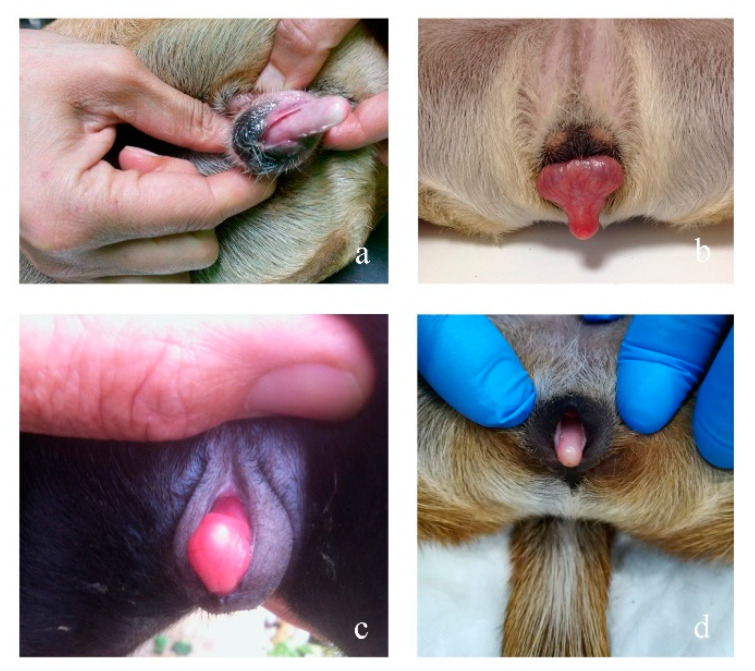
Abnormal clitoris (Megaclitoris) in the four analyzed cases: (**a**) Case 1; (**b**) Case 2; (**c**) Case 3; (**d**) Case 4.

Four dogs have been included in this study. All the cases were submitted to clinical evaluations due to the abnormal morphology of their external genitalia, in particular they all showed female phenotype, abnormal ano-genital distance and enlarged clitoris with penis bone (Figure 1). Except Case 1, the animals have all undergone surgery to remove the abnormal internal genitalia and to recreate the normal aspect of the external ones. 

### 2.3. Morphological Analyses 

Morphopathological analyses were performed on Case 2, Case 3 and Case 4. The animals underwent surgery for the removal of internal genitalia as requested by their owner. Samples from testis and reproductive tissue were collected and fixed in neutral-buffered 10% formalin solution (code no. 05-01007Q, Bio-Optica, Milan, Italy), dehydrated and embedded in paraffin (code no. 06-7920, Bio-Optica, Milan, Italy). Tissue sections were stained with hematoxylin-eosin (HE) and examined by light microscopy.

### 2.4. Cytogenetic Analyses 

Cell cultures for chromosome isolation were set up according to Macrì et al. [24] with limited modifications. Blood lymphocytes were cultured in RPMI medium (BE12-702F, Lonza, Basel, Switzerland) with lectin from phaselous vulgaris (code no. L1668, Sigma-Aldrich, St. Luis, MO, USA) and 10% of FBS, Australian origin, (code no. 10099141, GIBCO) for about 72 h at 37.5 °C (Macrì et al., 2012). Two types of cultures, with and without 5-BrdU, were setup. An amount of 20 μg/mL of 5-BrdU (code no. B5002, Sigma-Aldrich, St. Luis, MO, USA) and H33258 (40 μg/mL) (code no. B1155, Sigma-Aldrich, St. Luis, MO, USA) were added to the latter 5 h before harvesting. Colcemid (code no. L0040, Microtech srl, Naples, Italy) was added 1 h before harvesting to all cultures. A hypotonic treatment with 0.075 M KCl and three fixations with Carnoy’s fixative were performed. Cell suspensions were used to prepare slides that were allowed to dry and then stained for conventional and R-banding. From slides with Acridine Orange staining treated for both conventional and R-banding techniques, 200 and 10 metaphases were examined, respectively. Karyotypes were arranged according to Switonski et al. [25].

### 2.5. Molecular Analyses

DNA was extracted from whole blood with Wizard^®^ Genomic DNA purification kit (Promega). To verify the presence/absence of SRY gene, the coding region was amplified in all for cases as well as AMELX/Y region that was used as control [26,27] Finally, in all 4 subjects, the entire coding region of the RSPO1 gene was amplified and sequenced as reported in De Lorenzi et al. [26]. The primer sequences are reported in Table 1.

### 2.6. qPCR Analyses

qPCR was performed on the same DNA samples with SYBR Green (Invitrogen 11733-038) to test *RevSex* region and *Enh3* for copy number alterations, using *Bgrl2* as control. The comparative CT method (ΔΔCT method) was used to perform an evaluation of the copy number present in the genome of analyzed dogs. All primers used in qPCR experiments are reported in Table 1.

### 2.7. Array-CGH Analyses

Array-CGH analyses was performed using a custom Agilent dog CGH microarray K133. All analyses were performed as reported in De Lorenzi et al. [28]. DNA extracted from a single female animal was used as a control. 

## 3. Results

### 3.1. Clinical Findings

Table 2 show data about the four analyzed cases.

### 3.2. Morphological Analyses

#### 3.2.1. Case 1

Ultrasound examination revealed the presence of two ovotestis-like structures in the abdominal cavity, caudally to the kidneys, while hormonal analyses showed a testosterone value of 3.45 ng/mL (normal value for a castrated dog was <0.5 ng/mL) confirming the presence of secreting testicular tissue. The owner did not agree to surgical removal of the gonads, thus morphological analyses were not performed. 

#### 3.2.2. Case 2

**Figure 2:**
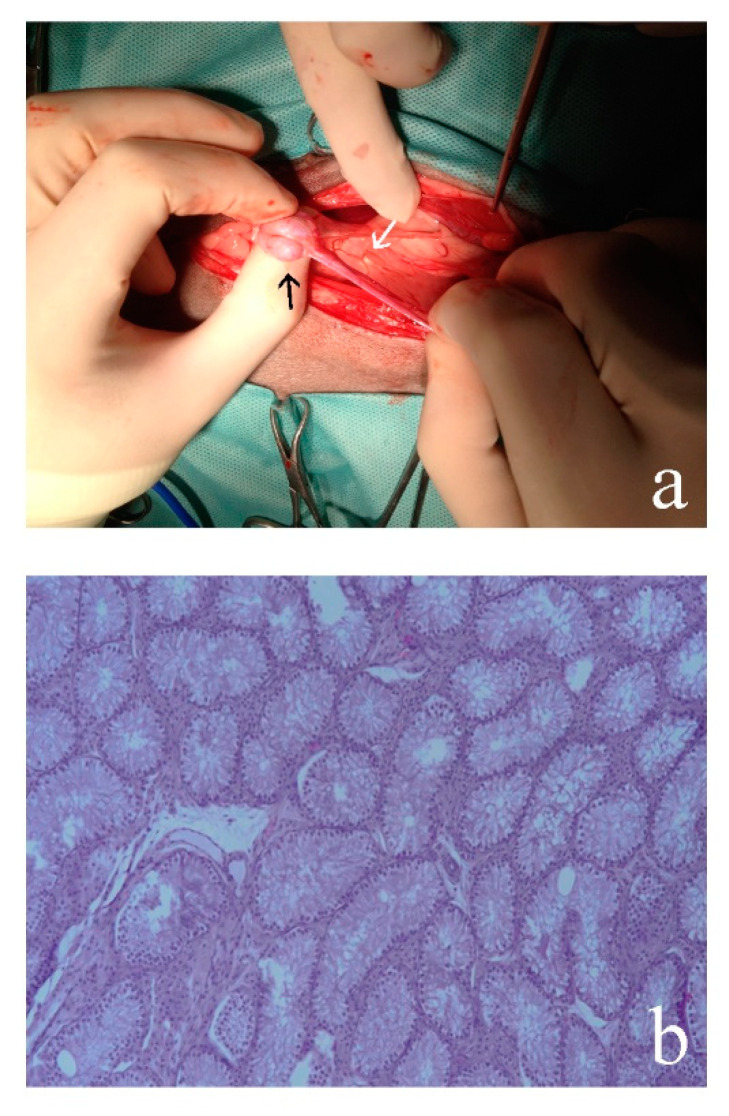
(**a**) Gonadectomy of Case 2 in which atrophic testis (black arrow) with uterine horn (white arrow) are showed; (**b**) histology of the atrophic testes in which is evident the structure of the tubules covered by Sertoli cells and without germ lines (20×) (see also supplementary Figure S1 for comparison). Hematoxylin–eosin (HE) staining.

Both gonads were atrophic testes (Figure 2) with tubules lacking germ lines and covered by Sertoli cells. At the edge of the gonads, a well-shaped and developed epididimus is evident. No ovarian follicle sketches were present. The uterus is well structured, delimited by a regular endometrium and containing neutrophils granulocytes, interspersed with necrotic debris (early-stage pyometra). At the extremity of the uterus there is an empty rounded structure of small size with a thick muscular wall and a normal epitelium that is compatible with a deferens vas.

#### 3.2.3. Case 3

**Figure 3:**
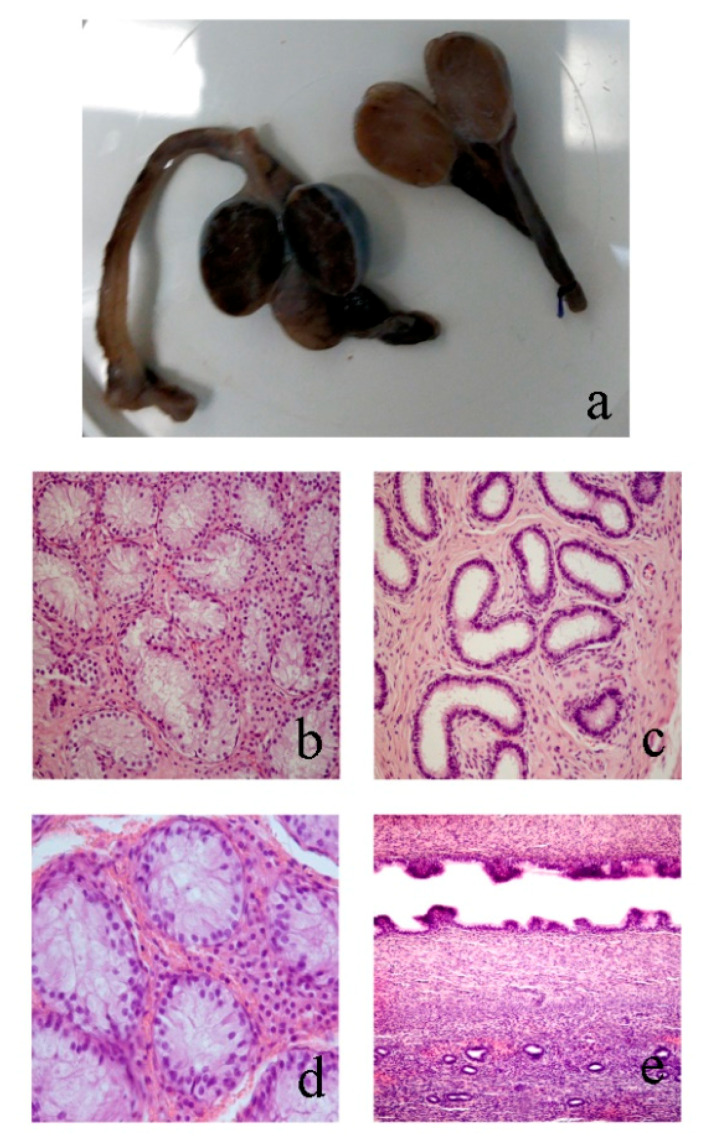
Case 3 (**a**) transversal sections of both the removed gonads; (**b**) section of the inguinal testis in which seminiferous tubules without spermatogenesis and many Leydig cells in the interstitium are evident (20×); (**c**) section of the epididymis in which empty tubules lined with columnar epithelium are shown (20×); (**d**) abdominal testis in which seminiferous tubules lined by Sertoli cells without germ cells are supported by interstitium with a hematic component and many Leydig cells (40×); (**e**) tubular structure with a lumen lined by simple epithelium forming short papillae and a dense fibrous layer with glandular structures are shown (20×). Hematoxylin–eosin staining.

The inguinally cryptorchid gonad had an ovoid shape and was connected to a tubular structure. One of the structures was referable to testis and the other to epididymis. The abdominally located gonad was smaller than the inguinal one and connected to a tubular structure which was similar to a hypoplastic uterine horn. Both gonads exhibited a testicular structure (Figure 3a), characterized by seminiferous tubules lined by Sertoli cells without germ cells. Interstitial tissue was abundant and many Leydig cells were present (Figure 3b). The epididymis was present in either gonad and showed empty tubules lined with columnar epithelium (Figure 3c). The abdominally located gonad showed a strong hematic component diffused in the interstitial tissue (Figure 3d). The tubular structure expressed features similar to a uterus with a lumen lined by simple columnar epithelium with an undulating pattern forming short papillae. Underneath the epithelium, a dense interstitial layer surrounded the glandular structures (Figure 3e).

#### 3.2.4. Case 4

**Figure 4:**
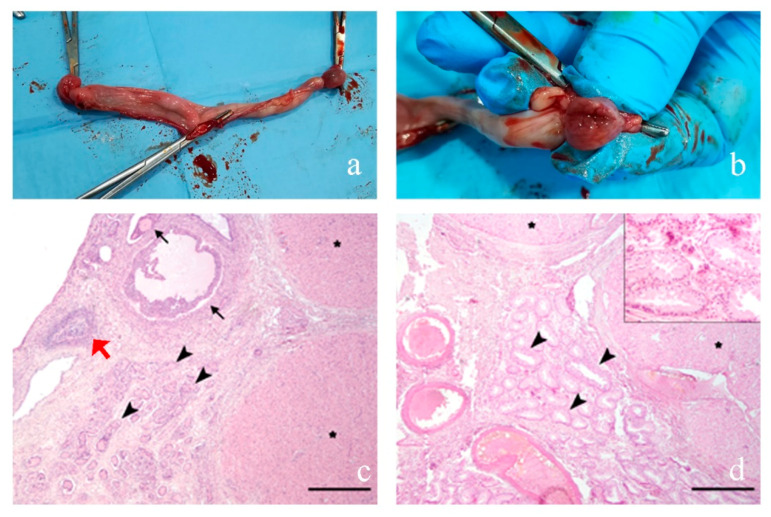
Genital apparatus surgically removed in Case 4. (**a**) A well-developed uterus and (**b**) section of gonads; (**c**) ovarian tissue composed of numerous follicles in various stages of development (black arrows), rare atretic follicles (red arrow) associated with few corpora lutea (asterisks) and, more deeply located, curvy seminiferous tubules (arrowhead), supported by abundant ovarian stroma; (**d**) testicular tissue composed of curvy seminiferous tubules (arrowhead) associated with two corpora lutea (asterisk) supported by a moderate amount of dense fibrous stroma with medium- and large-sized blood vessels. Insert: seminiferous tubules are lined by one layer of Sertoli cells extending from the basement membrane and protruding into the lumen and diffusely lack germ cells (hypoplasia). Interlaced with seminiferous tubules there are rare groups of Leydig cells. Hematoxylin–eosin (HE) staining. Scale bars = 500 μm.

Macroscopically, both gonads were ovoidal in shape (Figure 4a,b) and 2.5 × 1.5 × 1.5 cm in size. Histologically, both samples were characterized by gonadal tissue rimmed by a line of cuboidal to flattened surface epithelium and composed of a cortical zone of ovarian tissue surrounding a medullary zone of testicular tissue. The ovarian tissue was composed of numerous follicles in various stages of development associated with few corpora lutea, rare atresic follicles and scattered, deeper located, granulosa cell cords, supported by abundant densely cellular stroma (ovarian stroma) (Figure 4c). The testicular tissue was composed of curvy tubules lined by one layer of Sertoli cells extending from the basement membrane and protruding into the lumen diffusely lacking germ cells (hypoplasia). Interlaced with hypoplastic tubules, a moderate number of round to oval, foamy cells (consistent with interstitial cells) were supported by loose fibrovascular stroma (Figure 4d). The uterus was histologically normal.

### 3.3. Cytogenetic Analyses 

**Figure 5:**
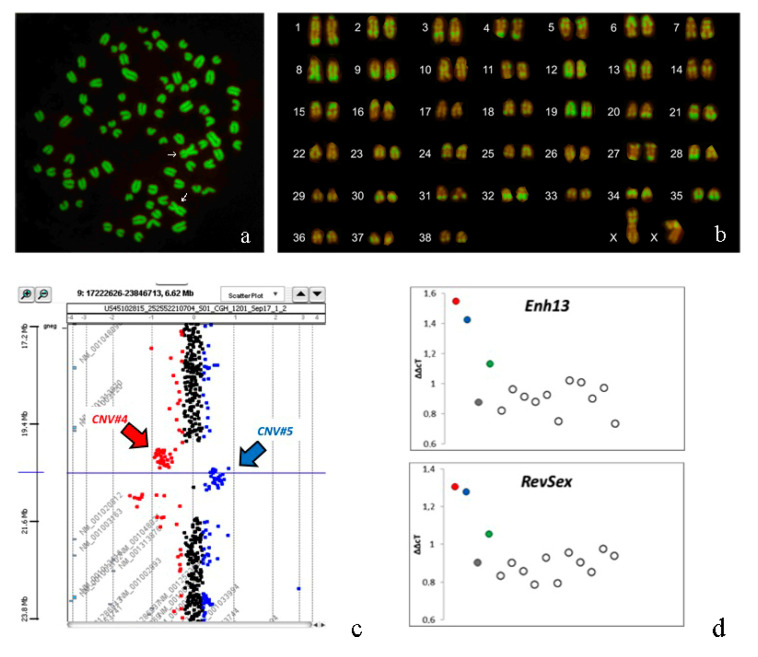
(**a**) Metaphase plate with 2*n* = 78; XX of Case 3 (arrows indicate X chromosomes); (**b**) R-banding (RBA) karyotype of Case 3; (**c**) CGH-array profile regarding copy number variation (CNV) number 5 and 4 identified in Case 1 (Case 2 and 4 show the same CNV profile). The points above indicate the number of copies of the region tested. Black: region present in two copies; red region with fewer copies than normal (deletion); blue: region with more copies than normal (duplication). The x axis indicates the genomic position on chromosome 9 of the probes analyzed. The x-axis indicates the log ratio identified after the CGH analysis; (**d**) qPCR results for RevSex and Enh13 regions in the 4 cases and 11 controls. The dots show the comparative CT method (ΔΔcT) values obtained in the 11 control dogs and for the 4 DSD dogs (Case 1: red; Case 2: blue; Case 3: grey and Case 4: green).

In all the cases, the analysis of conventional banded chromosome showed a normal 2*n* = 78; XX chromosome constitution. No numerical abnormalities were found for sex chromosomes. Karyotyping of R-banded metaphases for all the cases showed a normal chromosome composition and the absence of any kind of aberration for all the cell clones (Figure 5a,b). 

### 3.4. Molecular Analyses

All cases were SRY-negative and no AMELY genes were detected, confirming the absence of the Y chromosomes (not shown). Furthermore, the sequencing of the coding region of the RSPO1 gene showed no mutation in all four cases.

### 3.5. Array-CGH Analyses

The results obtained are shown in Table 3. In the four cases, we identify 11 CNVs and one of them, number 5 (Figure 5c), seems to be included in SOX9 promoter region.

### 3.6. qPCR

The Array-CGH analysis revealed several CNVs and one of these proved to be particularly interesting, being located in the 5 region in Sox9. This CNV contains regions homologous to *RevSex* and *Enh13*, regions that have been characterized as very important in controlling Sox9 expression. We tested, with a qPCR, the copy number state of these two genomic positions deemed interesting. The results are reported in Figure 5d and they confirm findings as observed in the CGH-array analyses.

## 4. Discussion

The four cases analyzed for this study combine a female aspect with a certain degree of masculinization for their phenotype explained by the presence of testicular tissue proved in all the subjects. 

Cases 2 and 3 are very interesting because they are from the same breed and both with one abdominal and one inguinal gonad. In both cases, the removed gonads showed a testicular morphology and complete absence of ovarian tissue. The lack of the ovotestis confirms they are XXSR males and not true hermaphrodites. Differently from other XXSR males, described in the literature and cited by [29], prostate was completely absent, and there was an abnormal vulva with an enlarged clitoris containing a bony base. This condition is the most frequently observed in French Bulldogs [30,31].

Histological analysis showed that Case 4 is different from the previous, in fact, the removed gonads showed both ovarian and testicular parenchyma, thus they can be classified as ovotestis. Ovotestis are often composed of ovarian tissue with numerous follicles, mostly at stages of degeneration and, testicular tissue with hypoplastic seminiferous tubules [32]. 

The testicular tissue found in the abnormal gonads, even without sperm production, as observed in the cases described, produces two hormones: testosterone from the interstitial cells and anti-Müllerian hormone from Sertoli cells, which act during early gestation to masculinize the developing mesonephric and paramesonephric ducts, tubules, and the external genitalia [33]. This mechanism explains the abnormal morphology of the external genitalia observed in these cases.

Cytogenetic analyses showed a XX karyotype in all four cases that, with the absence of the SRY gene, confirms the diagnosis of XX SRY-negative subjects. For this analysis, the AMEL gene was used as an additional control. This gene, in fact, is located on both sex chromosomes (X and Y) but shows a different length and for this reason is used for sexing and to check the presence Y chromosome [26]. Dog represents an excellent animal model in that the same phenotype (generally attributable to the presence of testicular tissue in the presence of a XX karyotype and absence of the SRY gene) is present, but very rare, in humans. The analysis of these subjects can help the identification of alterations in genetic factors capable of mimicking the function of the SRY gene in activating the male development pathway.

**Figure 6:**
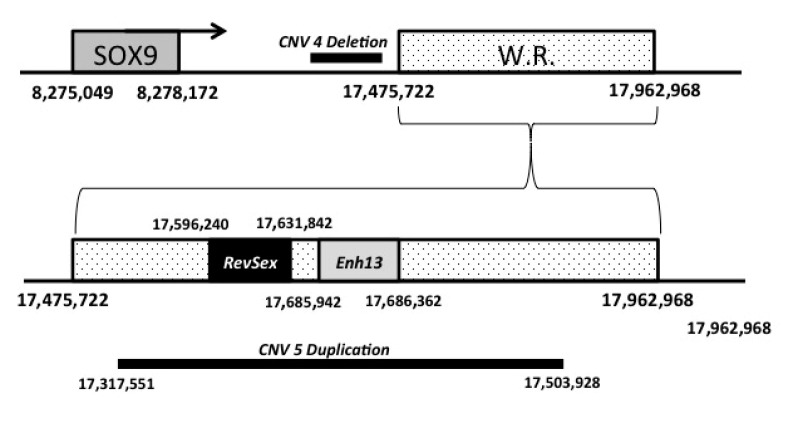
Schematic representation of the chromosome 9 genomic region where the SOX9 gene, *RevSex*, *Enh13*, Del CNV #4 and Dup CNV #5 are located. W.R. = Wrong Assembled Region.

From a genetic point of view, the most interesting result concerns the identification of a CNV involving the *SOX9* gene in three subjects, the CNV number 5 reported in Table 3. This CNV is identified as a duplication involving 458 Kb comprising the genomic region between base 17,503,928 and base 17,962,221 of chromosome 9 (CanFam3 genome assembly, Table 3 and Figure 5c). Apparently, this CNV is present in 3′ to the SOX9 gene (chr9: 8,275,049–8,278,172) and therefore it is unlikely that it will affect the regulation of this gene. However, it has recently been shown that the assembly reported in CanFam 3.1 of this particular chromosomal region is incorrect as shown by Rossi et al. [33]. In this work, it is shown that the chr9: 17,475,722–17,962,968 genomic region, reported in the assembly in 5 ‘to the sox9 gene, is probably positioned in 5′ to the SOX9 gene and that it contains both the *RevSex* region (chr9: 17,686,362–17,685,942) and the *Enh13* region (chr9: 17,596,240–17,631,842; Figure 6). Therefore the duplication identified in subjects 1, 2 and 4 would include both these regions probably positioned in the promoter region of the *Sox9* gene. This duplication was confirmed by *q*PCR analyses in subjects 1, 2 and 4 (Figure 5d). This result is very interesting, considering that duplication of one of these genomic regions can lead to XX SR in humans. It is not the first time that this genomic region has been involved in CNV: it had previously been identified as a deletion by Chen et al. [34] and by Mortlock et al. [35]. Nevertheless this CNV had already been described as a deletion and never as a duplication.

In all these three cases, this CNV is always associated with a deletion (CNV #4), but in this case the genomic fragment is well assembled and this CNV is 3′ to SOX9 gene (Figure 6); moreover, this genomic fragment was also previously reported to be involved in a CNV variation [34,35].

Regarding the other CNVs discovered, numbers 2, 3 and 8 were previously reported [34,36], while CNV 6 was reported by Mortlock et al. [35]. So it is certain that these CNVs represent polymorphisms present in the canine genome and in this particular situation, they are not associated with the observed DSD phenotype. The other CNVs identified (1, 7, 9, 10, 11, Table 3) have so far never been described. Among these, CNV number 1 and 9 were observed in all four samples and probably face a CNV in the control DNA. CNV #7, reported as homozygous deletion in three samples, contains DLA-DQA1 and DLA-DQB1 genes both belonging to major histocompatibility complex, class II gene family. CNV #10 contains CELA1 gene. This gene plays an important role in pancreas metabolism [37]. This genomic region includes also a region with similitude with human GALNT6 and BIN2 genes: both these genes are not involved in sex determination process [38,39]. Finally the genomic region involved in CNV #11 probably contains SPIN1 gene, a genetic factor involved in the meiotic spindle in oocytes [40].

## 5. Conclusions

In conclusion, we report in this paper the characterization of four new cases of the most important DSD: XXSR SRY and we report a duplication involving both *RevSex* and *Enh13* regions located 5′ to *SOX9* transcription starting site.

## Figures and Tables

**Table 1:** Primers used in PCR and qPCR.

Gene	Primers	Sequence (5′-3′)	Amp Size (bp)	Genome Pos ^1^
*Sry*	*Sry-F*	GCTGGGCGGAGAAATGAGTA	783	Not available
	*Sry-R*	CCAAGGTTTCCGGACTGTCA		
*Amelx/y*	*DSI-F*	ATAATGACAAAGAAAACATGAC	215/247	Chrx: 7,828,350–7,828,136 ^2^
	*DSI-R*	CTGCTGAGCTGGCACCAT		
*Rspo1*	*Rspo1-Ex1-f*	GCAGGCGTTAGCAAGAGC	297	Chr15: 4,862,841–4,863,138
	*Rspo1-Ex1-r*	ATCTGCAACGGTCATCACG		
	Rspo1-Ex2-f	AAGCACGTTCACGTTAGTCTTG	398	Chr15: 4,871,649–4,872,047
	*Rspo1-Ex2-r*	ACCAATGGGTCAAAGCACTC		
	*Rspo1-Ex3-f*	GTCACTCGGGCCTCCTCTA	478	Chr15: 4,873,791–4,874,269
	*Rspo1-Ex3-r*	GCAGAAAAGCTCGGAGACAA		
	*Rspo1-Ex4-f*	ACTGACACTGCCTCCAGCAT	480	Chr15: 4,874,276–4,874,754
	*Rspo1-Ex4-r*	CTGTTGTCTGCCAGCGTCT		
	*Rspo1-Ex5-f*	GGGGACCCTGAGACTGTGTA	399	Chr15: 4,875,173-4,875,572
	*Rspo1-Ex5-r*	TCCAGTTCCGTAAAGCTTCC		
*Enh13*	*Enh13-f*	GCAATGTGCACAGTTTCAGAG	118	Chr9: 17,686,202–17,686,320
	*Enh13-r*	TGAGGAATTAGAAGGCCATGA		
*RevSex*	*RevSex-Dog-F*	GACACTGTCCTGGGGAGAAA	100	Chr9:17,605,471- 17,605,570
	*RevSex-Dog-R*	TGAAGGCCAAGAGGCTAAGA		
*Bglr2*	*BGLR2-F*	GTGGAAGCCTGCAATTGTCT	203	Chr6: 734,406–734,609
	*BGLR2-R*	CCGTGAACAGGTGTAATGCT		

^1^ CanFam3.1. ^2^ Only position of *AmelX* gene is available.

**Table 2:** Clinical description of the four analyzed dogs.

Case Number	Breed	Age at First Clinical Evaluation	Phenotype	Surgical Findings
1	Staffordshire terrier	1Y	Female.	Ultrasound showed two ovotestis like structures in the abdomen caudally to the kidneys.
2	French bulldog	9M	Female. Presence of a little palpable mass in inguinal region	A gonad with a uterine horn was removed from the abdomen and another gonad was removed from the inguinal region
3	French bulldog	6M	Female. Presence of a little palpable mass in inguinal region	One gonad was in inguinal position and connected with a tubular structure. The other gonad was in the abdomen caudally to the kidney and was connected with a tubular structure that showed a fork linked to the vas deferens. Both structures ended in a uterine horn-like structure that lead directly into the vagina. The prostate was absent. Enlarged clitoris with penis bone protruded from an abnormal vulvae opening.
4	Mongrel	1Y	Female. 4.8 kg.	Two ovotestis like structures were found in the abdomen connected with uterine horns that merge in a uterine like structure ending in the vagina.

**Table 3:** CGH-array results.

CNV	CHR	CanFam3	END (bp)	Analyzed Subjects				
		START (bp)		SIZE (kb)	1	2	3	4
1	Chr 4	106,352	469,199	363	DEL	DEL	DEL	DEL
2	Chr 5	78,189,869	78,389,978	200		DEL	DUP	DEL
3	Chr 6	45,163,433	47,125,036	1962	GAIN	GAIN	GAIN	GAIN
4	Chr 9	16,906,864	17,317,551	411	DEL	DEL		DEL
5	Chr 9	17,503,928	17,962,221	458	DUP	DUP		DUP
6	Chr 9	38,978,944	38,995,409	16	DEL	DEL		DEL
7	Chr 12	2,191,427	2,270,973	80		DEL	DEL	DEL
8	Chr 23	20,508,926	20,725,281	216	GAIN		GAIN	
9	Chr 26	27,171,599	27,220,687	49	DELHO	DELHO	DELHO	DELHO
10	Chr 27	3,532,831	3,573,782	41	GAIN	GAIN		
11	Chr X	71,752,458	72,234,092	482	DELHO	DEL	DEL	

DEL: heterozygous deletion; DUP: heterozygous duplication; GAIN: duplication; DELHO: homozygous deletion.

## Supplementary Materials

The following are available online at https://www.mdpi.com/2076-2615/10/9/1667/s1, Figure S1: Microphotographs of normal seminiferous tubule from a control dog. Hematoxylin and Eosin stain (HE). In the lumen of the seminiferous tubule, adhering to the basement membrane, spermatogonia (thin black arrow) and Sertoli cells (white arrow) are evident. The more internal cell layers are composed of spermatocytes (thick black arrow) and spermatids (arrowhead) at different stages of maturation. In the interstitium some groups of Leydig cells are evident (asterisk).
