# Supplementary material for: Analysis of XX SRY-Negative Sex Reversal Dogs
Source: Animals (Basel). 2020 Sep 16;10(9):1667. doi: 10.3390/ani10091667 (PMC7552623; doi:10.3390/ani10091667)
Supplement: Supplementary file 1 [file animals-10-01667-s001.pdf]

Supplementary material

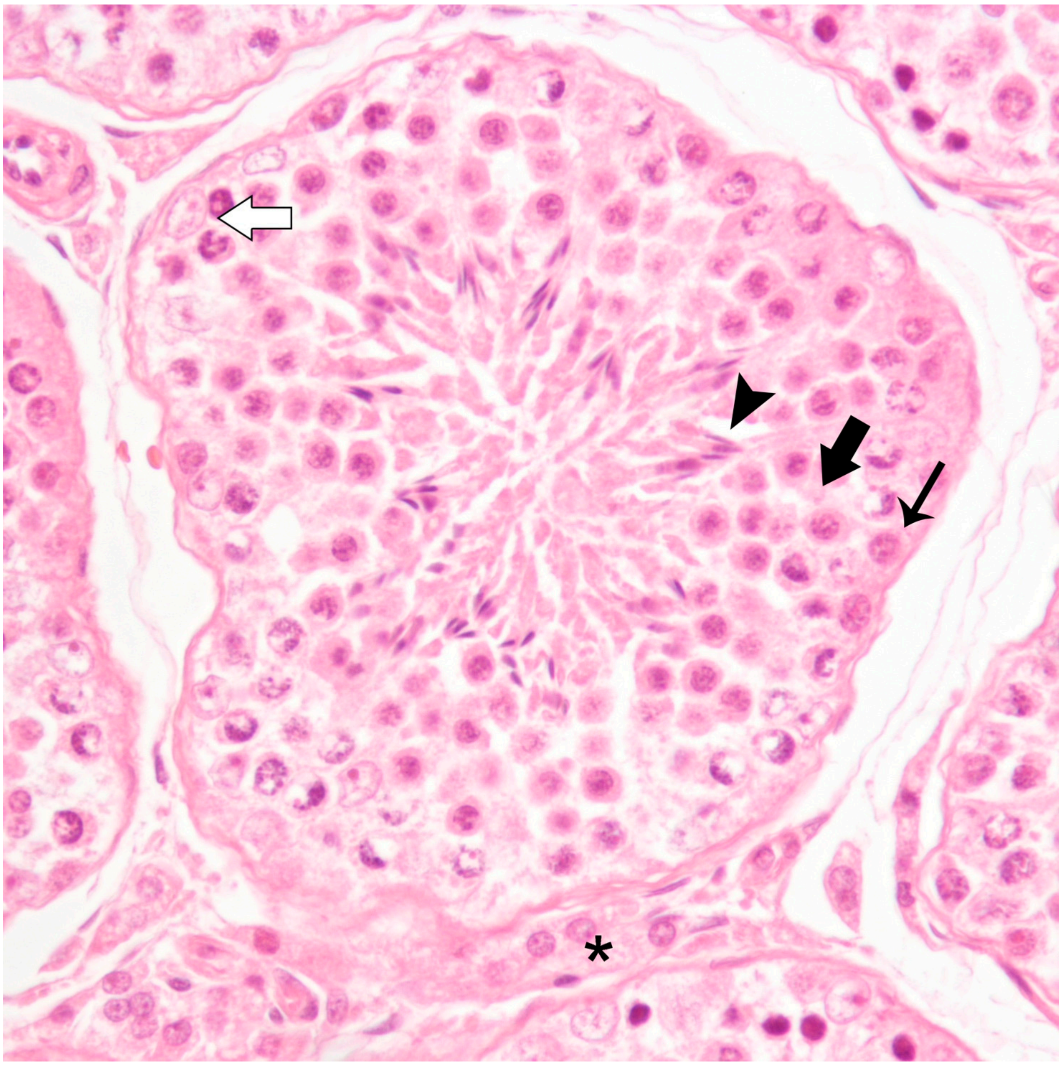

Figure S1. Microphotographs of normal seminiferous tubule from a control dog. Hematoxylin and Eosin stain (HE). In the lumen of the seminiferous tubule, adhering to the basement membrane, spermatogonia (thin black arrow) and Sertoli cells (white arrow) are evident. The more internal cell layers are composed of spermatocytes (thick black arrow) and spermatids (arrowhead) at different stages of maturation. In the interstitium some groups of Leydig cells are evident (asterisk).
